# Rib hump deformity assessment using the rib index in adolescent idiopathic scoliotics treated with full screw or hybrid constructs: aetiological implications

**DOI:** 10.1186/1748-7161-10-S2-S10

**Published:** 2015-02-11

**Authors:** Konstantinos C Soultanis, Nikolaos A Stavropoulos, Theodoros B Grivas, Konstantinos Tsiavos, Konstantinos Starantzis, Panayiotis J Papagelopoulos

**Affiliations:** 1 1st Department of Orthopaedics, University of Athens, Medical School, University General Hospital ATTIKON, Chaidari, Athens 12462, Greece; 2 Department of Orthopaedics and Traumatology, “Tzaneio” General Hospital of Piraeus, Tzani and Afendouli 1, Piraeus 18536, Greece

## Abstract

**Background:**

Review of literature reveals that in Idiopathic Scoliosis (IS) children, the post-operative rib hump (RH) correction using full transpedicular screw construct has never been compared to hybrid constructs, applying the Rib-Index (RI) method. Therefore the aim of this report is to study which of the above two constructs offers better postoperative Rib Hump Deformity (RHD) correction.

**Methods:**

Twenty five patients with Adolescent Idiopathic Scoliosis (AIS) were operated using full pedicle screw construct or hybrid construct. Sixteen underwent full screw instrumentation (group A) and nine an hybrid one (group B). The median age for group A was 15 years and for group B 17.2 years. The RHD was assessed on the lateral spinal radiographs using the RI. The RI was calculated by the ratio of spine distances d1/d2, where d1 is the distance between the most extended point of the most extending rib contour and the posterior margin of the corresponding vertebra on the lateral scoliosis films and d2 is the distance from the least projected rib contour and the posterior margin of the same vertebra. Moreover the amount of RI correction was calculated by subtracting the post-operative RI from the pre-operative RI.

**Results:**

Although within group A the RI correction was statistical significant (the pre-op RI was 1.93 and the post-op 1.37; p<0.001) and similarly in group B (the mean pre-op RI was 2.06 while the mean post-op 1.51; p=0.008), between group A and B the post-operative RI correction mean values were found to be no statistically significant, (p=0.803).

**Conclusion:**

Although the pre- and post-operative RI correction was statistically significant within each group, this did not happen post-operatively between the two groups. It appears that the RHD correction is not different, no matter what the spinal construct type was used. Provided that the full screw construct is powerful, the post-operative derotation and RHD correction was expected to be better than when an hybrid construct is applied, which is not the case in this study. It is therefore implied that the RHD results more likely from the asymmetric rib growth rather than from vertebral rotation, as it has been widely believed up to now. In 2013 Lykissas et al, reported that costoplasty combined with pedicle screws and vertebral derotation significantly improved RH deformity as opposed to pedicle screws and vertebral derotation alone. Another interesting implication is that the spinal deformity is the result of the thoracic asymmetry, implication in line with the late Prof. John Sevastikoglou’s (Sevastik’s) thoracospinal concept.

## Background

Idiopathic scoliosis is a 3D deformity of the trunk with detectable bony asymmetries and deformities, not only of the spine - the central axis - but also of the rib cage and other parts of the body. The thorax, like any other part of the skeleton, displays variations in dimensions and proportions [1]. The bony framework of the thorax changes so that the side to side width of the chest cavity exceeds progressively the anteroposterior length, while the ribs become structurally stronger as the child grows up [1-3]. Review of literature reveals that in IS the post-operative correction of the thoracic deformity after application of full transpedicular screw has never been compared to hybrid constructs, using the Rib-Index (RI) [4,5].

The aim of this report is to study whether there is a difference of the post-operative RHD correction between these two constructs.

## Methods and material

**Figure 1:**
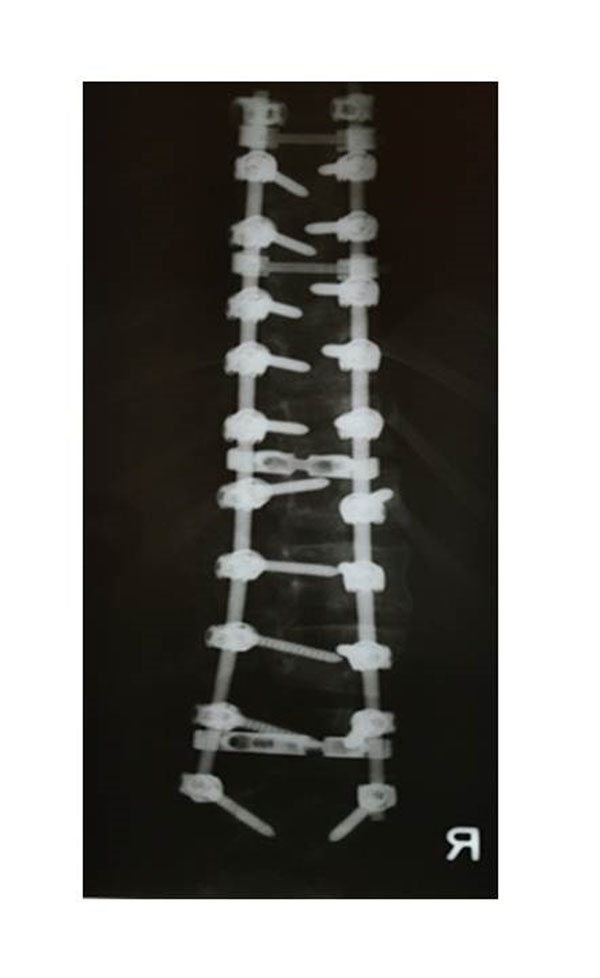
**Patient group A** The patients of this group were operated upon using a full pedicle screw construct.

**Figure 2:**
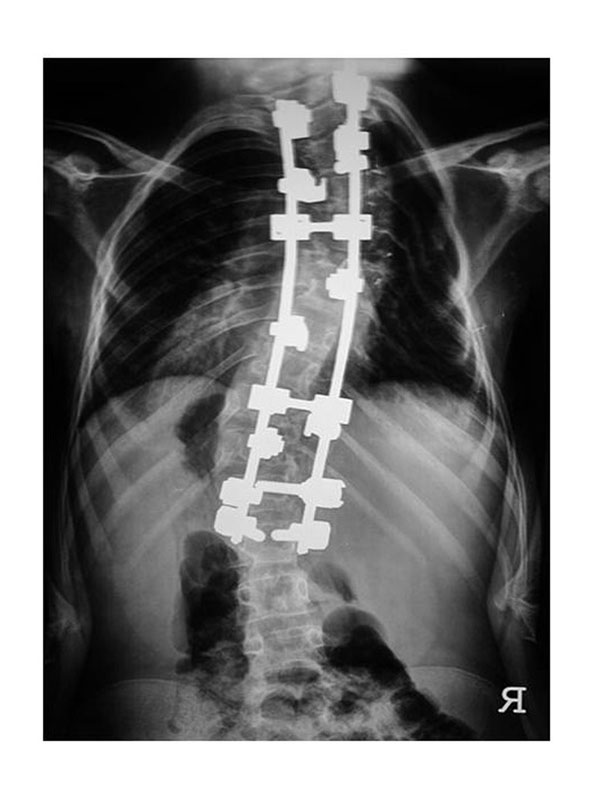
**Patient group B** The patients of group B were operated upon using an hybrid construct.

Sixteen patients with Adolescent Idiopathic Scoliosis (AIS) were operated using a full pedicle screw construct (group A) (Figure 1), while nine were treated using a hybrid construct (group B) (Figure 2). Fourteen out of the sixteen patients in group A had right and two had a left thoracic curve; seven curves were Lenke’s type 3C, six type 1A, two type 2A and one 6C. Among the 9 patients of the group B, 4 scoliotics had Lenke’s type 1A curves, 3 type 6C and 2 type 2A. The median age for group A was 15 years and for group B 17.2 years. The RHD was assessed on the lateral spinal radiographs using the RI, as it was described by Grivas et al 2002 [3]. Moreover the amount of RI correction was calculated by subtracting the post-operative RI from the pre-operative RI. Statistical analysis was performed using the Wilcoxon test, (IBM SPSSv.20 package). In order to access the data of the patients this study was approved by the Ethical & Scientific Committee for Clinical Research at "ATTIKON" Hospital.

## Results

**Figure 3:**
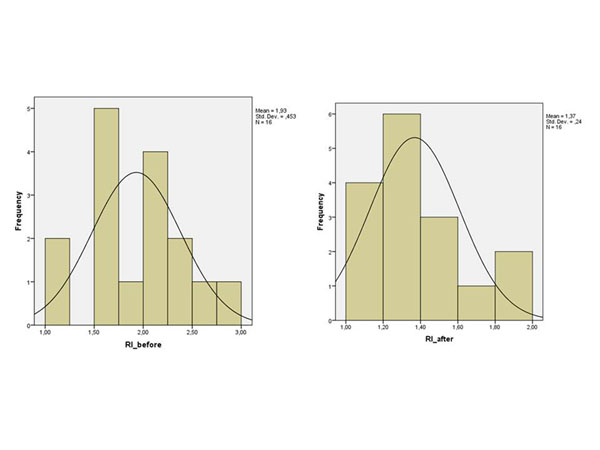
**Pre and Post-operative RI of the group A** The following charts show the pre- and post-operative RI of the patients of group A.

**Figure 4:**
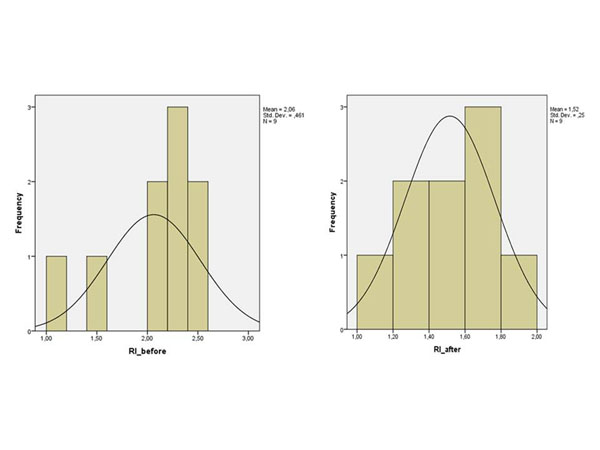
**Pre- and Post- operative RI of the group B** The following charts show the pre- and post-operative RI of the patients of group B.

**Figure 5:**
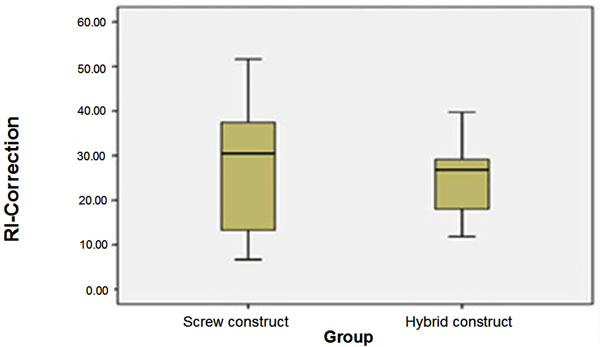
**Correlation of post-operative RI correction between groups A and B.** The correlation of the post-operative RHD correction between the two groups was not statistically significant different. The following boxplot depicts the lack of statistical significance.

In group A the mean pre-operative RI was 1.93 and the post-operative 1.37 (p<0.001) (Figure 3). Similarly in group B the mean pre-operative RI was 2.06, while the mean post-operative 1.51, (p=0.008) (Figure 4). However, between group A and B the post-operative correction RI mean values were not statistically significant (p=0.803) (Figure 5).

## Discussion

The scoliosis surgery techniques evolved from the Harrington era and the advent of new technologies proved to be promising. A challenging question is what type of surgical instrumentation offers better rib-hump correction: a full screw or an hybrid construct? The full screw construct is more powerful and therefore frequently used. However this study reveals that whatever the type of instrumentation used, there was no statistically significant post-operative difference in correction between the two groups, in terms of RHD. The RHD more likely seems to result from the asymmetric rib growth than from vertebral rotation.

There are certain limitations in this study that is its retrospective nature, the limited number of patients involved and the relative heterogeneity of the curve types.

## Conclusions

Although the pre- and post-operative RI correction was significant within the two studied groups of patients, none of the two constructs used offered better RHD correction. Provided that the full screw construct is powerful, the post-operative derotation and the RHD correction should have been better corrected than with an hybrid construct, yet this was not the case in this study. This discordance between the ribcage and the spine behavior after surgery could be attributed to a different scoliogenic course and this could have potential aetiological implications. It seems likely that the thoracic deformity contributes to RHD more than the potential vertebral rotation.

This is the extended abstract of IRSSD 2014 program book [6].

## Consent

The ethical & scientific committee of "ATTIKON" Hospital has approved the use of patients data and imaging respecting their privacy, for the purpose of the retrospective clinical study titled <Rib hump deformity assessment using the rib index in adolescent idiopathic scoliotics treated with full screw or hybrid constructs: aetiological implications>. Patients were informed prior medical services or admission to the University Hospital "ATTIKON" that imaging and other medical data could be used in teaching and academic purposes.

## Competing interests

There no competing interests to disclose.

## Authors' contributions

TBG: Conceived the idea of RI, drafted the text and searched the literature. NAS: Drafted the text, searched the literature. KCS: Reviewed the paper. All authors contributed their professional skills to the inclusions of the text. All authors have read and approved the final manuscript.

## Declarations

Publication charges for this collection were funded by IRSSD 2014 Sapporo.

This article has been published as part of *Scoliosis* Volume 10 Supplement 2, 2015: Research into Spinal Deformities 9: Short Papers from the IRSSD 2014 Meeting. The full contents of the supplement are available online at http://www.scoliosisjournal.com/supplements/10/S2.
